# Non-caloric artificial sweeteners modulate conjugative transfer of multi-drug resistance plasmid in the gut microbiota

**DOI:** 10.1080/19490976.2022.2157698

**Published:** 2022-12-16

**Authors:** Zhigang Yu, Ian R. Henderson, Jianhua Guo

**Affiliations:** a Australian Centre for Water and Environmental Biotechnology (Formerly AWMC), The University of Queensland, Brisbane, Australia; b Institute for Molecular Bioscience, The University of Queensland, Brisbane, Australia

**Keywords:** Antibiotic resistance, non-caloric artificial sweeteners, gut microbiota, conjugation, plasmid, reactive oxygen species, 16S rRNA

## Abstract

Non-caloric artificial sweeteners have been widely permitted as table sugar substitutes with high intensities of sweetness. They can pass through the intestinal tract without significant metabolization and frequently encounter the gut microbiome, which is composed of diverse bacterial species and is a pool of antibiotic resistance genes (ARGs). However, little is known about whether these sweeteners could accelerate the spread of ARGs in the gut microbiome. Here, we established an *in vitro* conjugation model by using *Escherichia coli* that carries chromosome-inserted Tn7 *lacI^q^-pLpp-mCherry* and plasmid-encoded *gfpmut3b* gene as the donor and murine fecal bacteria as the recipient. We found that four commonly used artificial sweeteners (saccharin, sucralose, aspartame, and acesulfame potassium) can increase reactive oxygen species (ROS) production and promote plasmid-mediated conjugative transfer to the gut microbiome. Cell sorting and 16S rRNA gene amplicon sequencing analysis of fecal samples reveal that the tested sweeteners can promote the broad-host-range plasmid permissiveness to both Gram-negative and Gram-positive gut bacteria. The increased plasmid permissiveness was also validated with a human pathogen *Klebsiella pneumoniae*. Collectively, our study demonstrates that non-caloric artificial sweeteners can induce oxidative stress and boost the plasmid-mediated conjugative transfer of ARGs among the gut microbiota and a human pathogen. Considering the soaring consumption of these sweeteners and the abundance of mobile ARGs in the human gut, our results highlight the necessity of performing a thorough risk assessment of antibiotic resistance associated with the usage of artificial sweeteners as food additives.

## Introduction

The emergence of antibiotic-refractory bacterial populations severely compromises the efficiencies of traditional antibiotic therapeutics. As a global threat to human health, antibiotic resistance causes over 700,000 global deaths per year.^1,2^ Bacteria can develop antibiotic resistance via horizontal gene transfer (HGT) to acquire antibiotic resistance genes (ARGs). HGT is a major force that drives the transfer of genetic mobile elements (e.g., plasmids) within and across phylogenetic genera. genera. ^3^ One of the most popular HGT pathways is plasmid-mediated conjugation, which involves close contact between donor and recipient cells and involves the transfer of plasmids carrying ARGs from the donor to the recipient. recipient. ^4^ Conjugation has been intensively studied using several conjugative plasmids. plasmids. ^5–7^

The gastrointestinal tract is a complex ecosystem in which microbial communities interact with each other, with their host, and with their environment. environment. ^8,9^ Gut microbiota consists of as many as 10^14^ bacterial cells belonging to over 1000 species-level phylotypes^10^ and plays a central role in the well-being of their host. ^11^ The multiple and inevitable interactions between these diverse phylotypes can be involved in ARG transfer under exposure to exogenous antibiotics. Antibiotics are well recognized to select antibiotic resistance and extensively shift human gut microbial composition. ^12–14^ Such alteration in microbiota composition also significantly correlates with individual diet styles. ^15–17^ Specifically, artificial sweeteners that have been permitted as table sugar substitutes and are being widely used in food and beverages can exert antimicrobial effects^18^ and alter gut microbiota composition. ^19^ These sweeteners can pass through the human digestive tract without being metabolized by the host^20^ and directly encounter the indigenous microbiota, thus modulating the interactions between the gut phylogenetic species.

In the gut system, the residing microbiota is a rich source of ARGs 21, and conjugation has been found as a common event among them. ^22,23^ In particular, plasmid-encoded ARGs can be transferred between commensal and pathogenic bacterial species. species. ^24^ Our previous work has reported that compared to table sugars (sucrose and glucose), artificial sweeteners such as saccharin, sucralose, aspartame, and acesulfame potassium could promote horizontal transfer of plasmid-encoded ARGs in single bacterial strains. ^25,26^ However, little is known about whether the spread of ARGs in the gut microbiota could be promoted by these sweeteners. Considering the substantial application of artificial sweeteners in food industry (over 117,000 metric tons globally consumed per year^27^) and the soaring consumption in people’s daily lives, it is necessary to further explore the roles of artificial sweeteners in the spread of antibiotic resistance in the gut microbiota, over the post-antibiotic era.

Here, we experimentally established an *in vitro* plasmid-mediated conjugation model by using mice gut microbiota as the recipient and comprehensively investigated the capability of four commonly used artificial sweeteners (saccharin (SAC), sucralose (SUC), aspartame (ASP), and acesulfame potassium (ACE-K)) to increase ARG transfer at gut relevant concentrations. To unravel the plasmid permissiveness of gut microbiota at the genus level, we employed high-throughput cell sorting and 16S rRNA gene amplicon sequencing techniques. In addition, we further validated the phenomenon by using the a nosocomial pathogen *Klebsiella pneumoniae,* which commonly causes lung infection and can also be detected in the intestinal tract^2828,29^ as the recipient. Intracellular reactive oxygen species (ROS) production, a critical contributor involved in the conjugation process^29,30^ was measured from both the donor and the initial fecal community treated with the sweeteners. Together, our results show that the tested four artificial sweeteners can significantly promote conjugative plasmid permissiveness among gut microbial communities and can also broaden such permissiveness to human pathogens.

## Results

### Non-caloric artificial sweeteners promote conjugative transfer in gut microbiota

**Figure 1.**
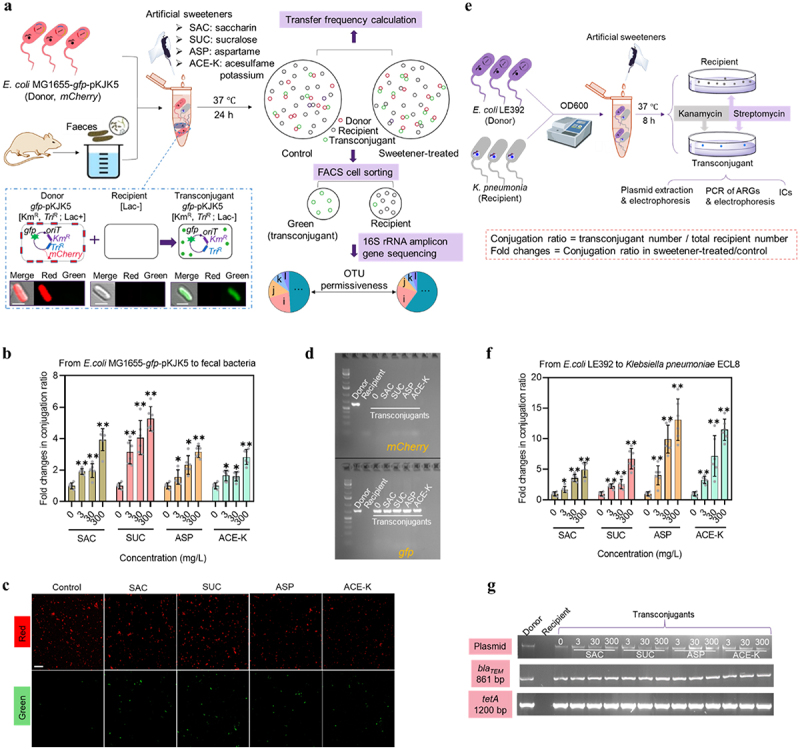
A *In vitro* conjugation model with mice fecal bacteria as the recipient. The donor *E. coli* K-12 MG1655 carrying *gfp*-encoded plasmid pKJK5 and fecal bacteria were mixed and incubated at 37°C for 24 h until a series of analyses such as detection of transconjugant and recipient by flow cytometry, confocal imaging, cell sorting (FACS), and 16S rRNA gene amplicon sequencing. The strains’ phenotypes used for mating, detection and sorting were indicated in brackets. All scale bars in cell images are 2 *μ*m. b Fold changes in conjugation ratio between *E. coli* MG1655 and gut bacteria communities, under exposure to 0, 3, 30 and 300 mg/L of the four sweeteners (*N* = 6). c Gel electrophoresis images of PCR assayed *mCherry* and *gfp* extracted from *E. coli* MG1655, fecal bacteria and FACS-sorted transconjugants. d Confocal microscopic images of plasmid transfer from the *E. coli* MG1655 donor to fecal bacteria community in the absence or presence of artificial sweeteners (at 300 mg/L). The detected red or green spots were confirmed with bacterial size by higher magnifications. All scale bars are 20 *μ*m. e Conjugation assay by using *K. pneumoniae* ECL8 as the recipient. The donor *E. coli* K-12 LE392 that carries RP4 plasmid and the recipient *K. pneumoniae* ECL8 were mixed together and were handled following the same procedure as *in vitro* model. The successful transfer of plasmid from the donor to the recipient was verified by plasmid extraction and electrophoresis, PCR assays and/or ICs against corresponding antibiotics. f Fold changes in conjugation ratio between *E. coli* LE392 (containing RP4 plasmid) and *K. pneumonia* ECL8, under exposure to 0, 3, 30 and 300 mg/L of the four sweeteners (*N* = 6). g Gel electrophoresis images of RP4 plasmid and PCR assay of *bla_TEM_* and *tetA* from *E. coli* LE392, *K. pneumonia* ECL8 and transconjugants. Significant differences between individual sweetener-treated groups and the control (0 mg/L of sweeteners) were tested with Independent-sample *t* test and the Bonferroni correction, * *p* < .05 and ** *p* < .01.

To study whether artificial sweeteners could promote the horizontal transfer of ARGs among gut microbiota, we conducted conjugation assays by using mice feces as the recipient. We used the donor *E. coli* K-12 MG1655 that was chromosomally labeled with *mCherry* and carried *gfp*-labeled pKJK5 plasmid. Conjugation events were detected as green-fluorescent cells counted by flow cytometer (Figure 1a). The mating method was established to screen out real green-fluorescent singlet (expressed by *gfp*) from the mating system (Figure S1). Spontaneous conjugation events were detected at ~1 in 8000 (1.2 × 10^−4^ ± 2.3 × 10^−5^) of the initial fecal bacterial cells in the absence of artificial sweeteners. This conjugation ratio significantly increased in a concentration-dependent manner once the mating system was exposed to artificial sweeteners (Figure 1b). For example, at 300 mg/L, SAC, SUC, ASP, and ACE-K induced 3.9- (*p* = 2.0 × 10^−6^), 5.3- (*p* = 1.2 × 10^−7^), 3.2- (*p* = 1.7 × 10^−7^), and 2.8-fold (*p* = 6.0 × 10^−6^) increases in plasmid transfer among fecal bacteria. This increase in conjugative transfer was supported by the confocal microscopic images (Figure 1c), showing that compared to the control, the number of green-fluorescent cells in thecreased in the presence of artificial sweeteners (at 300 mg/L). The enhanced conjugation was not due to the associated osmolarity and solution pH (Figures S2 and S3). In addition, the transconjugants were sorted out from the mating system by using FACS technique. Compared to the recipient, FACS-sorted transconjugants carried *gfp* gene that was originated from pKJK5 plasmid (carried by the donor), indicating the successful conjugative transfer from the donor to fecal bacteria (Figure 1d).

In the pure culture model of conjugation between *E. coli* LE392 carrying RP4 plasmid and *K. pneumoniae* ECL8 (Figure 1e), the conjugative transfer ratio also showed a dose-dependent increase in the presence of artificial sweeteners, with the largest increase of 4.9- (*p* = 3 × 10^−6^), 6.7- (*p* = 4 × 10^−6^), 13.1- (*p* = 9 × 10^−6^), and 11.5- (*p* = 8 × 10^−6^) fold at 300 mg/L of SAC, SUC, ASP, and ACE-K, respectively (Figure 1f). This increase was due to the increase of colony number on the transconjugants plates (Figure S4) but not to the total number of viable recipient cells (Figure S5). The successful conjugation between the donor and recipient was confirmed by the plasmid DNA extraction and PCR assays of target genes (two ARGs *bla_TEM_* and *tetA* from RP4 plasmid) (Figure 1g), as well as the ICs measurement (Figure S6).

Collectively, artificial sweeteners can significantly enhance the conjugative transfer of plasmid DNA among gut bacteria communities. Such phenomenon is also observed in the human pathogen.

### Non-caloric artificial sweeteners trigger ROS overproduction

Using fluorescence-based analyses, our previous results indicate that the activation of stress response and the increased cell membrane permeability that are involved in conjugation process can be due to the formation of an intracellular reactive species.^26^ We have previously used 2’,7’-dichlorofluorescein diacetate, which has been widely reported *in vitro* selectivity for highly total reactive species, but it cannot distinguish species such as peroxyl and nitric oxide (NO). Here we used a diverse panel of fluorescent reporter dyes, cellular ROS/RNS 3-Plex detection mix, to detect the production of total ROS, peroxyl and NO radicals in the donor and recipient cells exposed to the above sweeteners.

**Figure 2.**
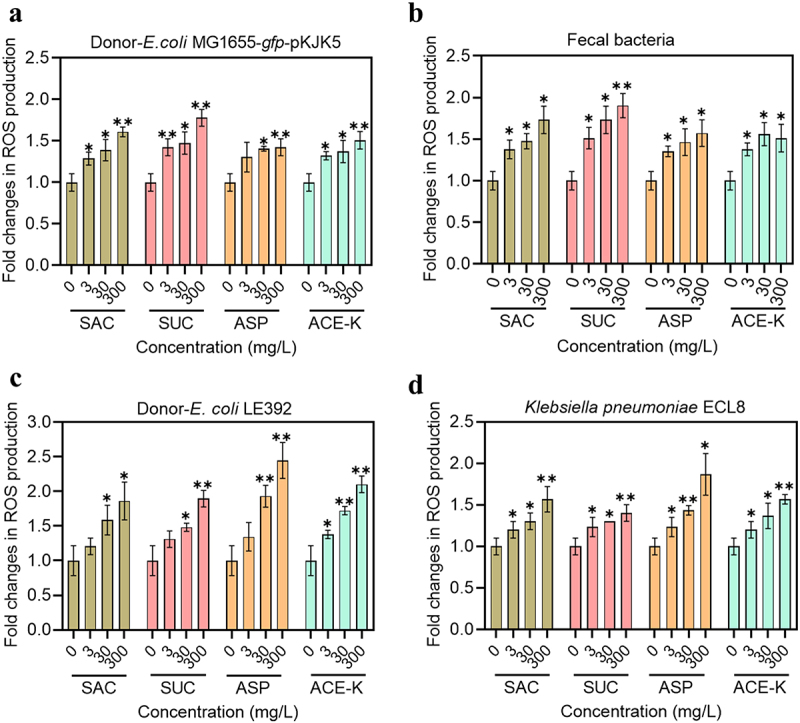
Generation of reactive oxygen species (ROS) induced by the treatments with artificial sweeteners at different concentrations. **a** Fold changes in ROS production in the donor strain *E. coli* MG1655 (*gfp*-pKJK5); **b** Fold changes in ROS production in mice fecal bacteria as the recipient. **c** Fold changes in ROS production in the donor strain *E. coli* LE392 (RP4); **d** Fold changes in ROS production in the recipient strain *K. pneumonia* ECL8. Significant differences between individual sweetener-treated groups and the control (0 mg/L of sweeteners) were tested with Independent-sample *t* test and the Bonferroni correction, * *p* < .05 and ** *p* < .01.

Notably, the tested four artificial sweeteners significantly increased intracellular ROS production in all mating systems, in a dose-dependent manner (Figure 2). For *in vitro* conjugation model, ROS production in the donor *E. coli* MG1655 and the initial fecal community was increased 1.4- to 1.8-fold (*p* < .007) and 1.5- to 1.9-fold (*p* < .031) by artificial sweeteners at 300 mg/L, respectively. Consistent with the highest conjugation ratio induced by 300 mg/L of SUC, the largest fold increases in ROS production from both donor and the initial fecal community were also induced under the same condition (Figure 2a, b). As well, peroxyl overproduction was significantly induced (Figure s7a, b). For NO radicals, we did not detect any significant increase from the mating system ((*p* > .102; Figure S8). For pure culture conjugation model, artificial sweeteners at 300 mg/L increased 1.9- to 2.5-fold (*p* < .013) and 1.4- to 1.9-fold (*p* < .016) ROS production in *E. coli* LE392 (donor) and *K. pneumonia* ECL8 (recipient), respectively, with the largest fold increases in ROS production induced by ASP (Figure 2c, d). Peroxyl radical was also significantly overproduced among the two strains (Figure S7c, d). Considering that in pure culture model, the condition for the highest fold increase in conjugative ratio was also the same (300 mg/L of ASP) as that for the highest ROS production, these results collectively suggest that ROS plays a critical role in the conjugative transfer process.

### Effect of non-caloric artificial sweeteners on the diversity in transfer host range

**Figure 3.**
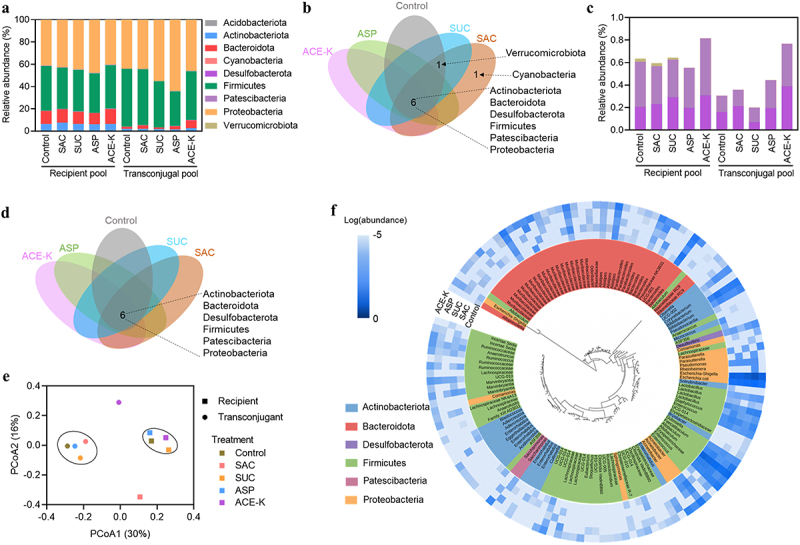
Diversity in transfer host range of mice fecal microbiome under exposure to artificial sweeteners. **a** Phylum-level relative abundance of the recipient and transconjugant pools from mice feces (the control and artificial sweetener-treated groups) after 24-h conjugation assay. **b** Venn diagram at phylum level of the recipient pools in the absence or presence of artificial sweeteners. **c** Relative abundance of rare taxa (relative abundance < 1%) from the recipient and transconjugant pools with or without artificial sweeteners treatment. **d** Venn diagram at phylum level of the transconjugant pools in the absence or presence of artificial sweeteners. **e** Bray-Curtis PCoA of the recipient and transconjugant pools at the genus level. **f** Phylogenetic tree showing the relative abundance of all 134 identified OTUs from transconjugant pools. Background colors of OTUs indicate different phylogenetic groups. The periphery blue heatmap-circle indicates the log-relative abundance of each OTU in the transconjugant pools.

To gain insights into whether artificial sweeteners could shift the diversity in transfer host range of mice fecal microbiome, we did cell sorting and 16S rRNA gene amplicon sequencing, and performed microbial profiling of both recipient and transconjugant communities in the absence or presence of artificial sweeteners. Across all the recipient pools, 8 phyla of taxa were identified, based on their relative abundance (Figure 3a). The dominant taxa from all the recipient pools consisted of Proteobacteria (α, γ) (relative abundance > 42.6%), Gram-positive phyla Firmicutes (relative abundance > 35.5%), Bacteroidota (relative abundance > 10.2%), as well as Gram-positive phyla Actinobacteriota (relative abundance > 6.0%) (Figure 3a). In addition to the dominant taxa, other phyla taxa such as Patescibacteria, Desulfobacteria and Verrucomicrobiota, as well as rare taxa can also be shared between the control and sweetener-treated groups (Figure 3b, c).

Across the transconjugant pools, 6 phyla of taxa were identified (Figure 3a). Multiple dominant taxa such as Proteobacteria, Firmicutes, Bacteroidota and Actinobacteriota that were identified in the recipient pools were also observed. It is expected that Proteobacteria could be the dominant transconjugant taxa because the donor strain *E. coli* MG1655 belongs to the Proteobacteria phylum and the conjugation within genera could be more easily proceeded than across genera.^26,32^ The relative abundance of Proteobacteria transconjugants may exhibit an increase in the presence of artificial sweeteners. For example, in the control group, the relative abundance of Proteobacteria transconjugant was 43.9%, while in ASP-treated group it was 64.0%. This indicates that artificial sweeteners preferably promoted conjugative transfer within bacterial genus. In addition, all 6 identified transconjugant phyla were shared between the control and the sweetener-treated groups (Figure 3d), suggesting that artificial sweeteners could not broaden such permissiveness at phylum level. Interestingly, a distinct variation of the ACE-K-exposed transconjugant microbiome from others was observed (Figure 3e; Figure S9a), indicating that ACE-K could shift the gut microbiomes as candidates for plasmid reception.

We further compared the microbial diversity of the recipient and transconjugant pools (at genus level) between the control and the treated groups by using the Shannon index and the Chao index. In the recipient pools, an increase in microbial diversity was only observed in the SAC-treated group compared with the control group (Figure S9b). Principal Coordinates Analysis (PCoA) also showed an inter-individual variation of the SAC-exposed recipient microbiome from other recipient pools (Figure 3e). Although 6 identified taxa from the recipient pools were identified in the transconjugant pools (Figure 3a, d), microbial community structure in the transconjugant pools was distinct from that in the recipient pools because of distinct clustering (Figure 3e). In the control transconjugant pool, up to 10-fold abundance of Bacteroidota declined when compared to its recipient pool. For the sweetener-treated transconjugant pools, the relative abundance of the most dominant phylum Proteobacteria increased, compared to their corresponding recipient pools (Figure 3a). This can be also supported by the higher values of Shannon index and Chao index in the recipient pools than those in the transconjugant pools (Figure S9). Moreover, results of the dominant Proteobacteria phylum and the shared 6 identified phyla in both recipient and transconjugant pools suggested that plasmid might be preferably permissible to the abundant phyla including phylogenetically both close (Proteobacteria) and distant (e.g., Firmicutes) phyla.

We also analyzed each transconjugant pool at genus level to unravel whether artificial sweeteners could modulate OTU permissiveness. Across all pools, the pKJK5 plasmid was permissible to 134 OTUs that were distributed over 6 phyla (Figure 3f). In the control group, 59 permissive genera were identified. By contrast, as much as 93 permissive OTUs were identified in the ACE-K-treated group. This suggests that artificial sweeteners are able to broaden the plasmid permissiveness among gut microbiota communities. The control group and the sweetener-treated groups shared over 27 permissive genera, accounting for the relative abundance >90% of each pool. These core permissive genera were mainly composed by *Escherichia, Allobaculum*, and *Erysipelotrichaceae* (Figure S10). As expected, *Escherichia* was the most abundant genus across transconjugant pools, further suggesting that plasmid could be more permissive between closely phylogenetic species. Moreover, several bacterial genera such as *Acinetobacter, Enterococcus* and *Staphylococcus* were identified in the transconjugant pools, highlighting the possibility of ARG transmission among gut pathogenic microbes. We also found that other bacteria including genera *Olsenella* and *Odoribacter* were solely identified in the sweetener-treated groups. This further suggests that artificial sweeteners could enhance conjugative plasmid permissiveness among gut pathogenic microbes.

### Effect of artificial sweeteners on ARG permissiveness among gut microbial community

**Figure 4.**
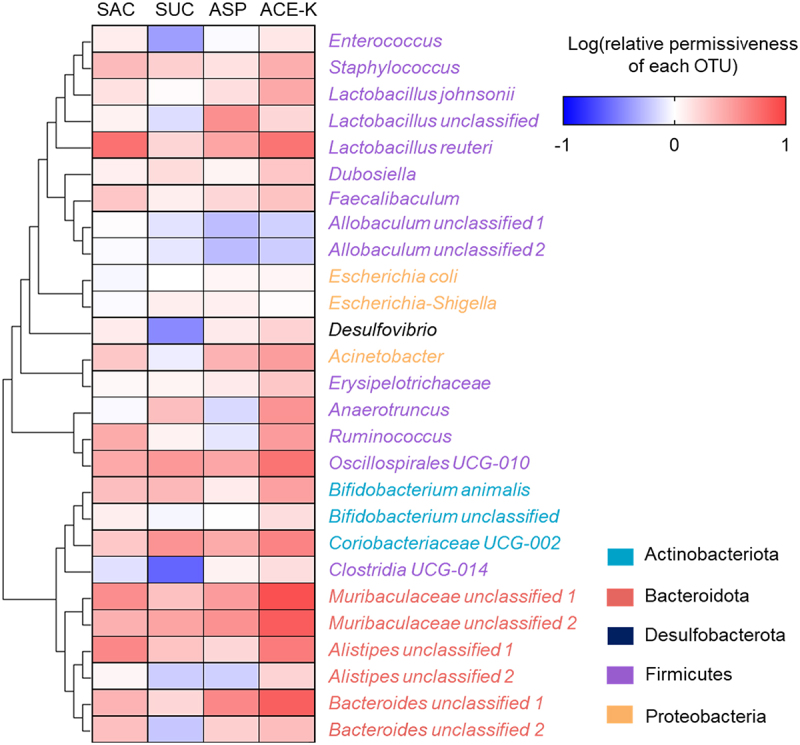
Heat-map analysis of plasmid permissiveness to the top 27 abundant OTUs in the transconjugant pools (relative abundance > 0.05%). The color of the font at right side of the heat-map indicates the taxonomy. Experimental treatments with 300 mg/L of artificial sweeteners were carried out.

To offer insights into what types of genera could be potential candidates as the recipients in the absence or presence of artificial sweeteners, we quantified the broad-host-range conjugative pKJK5 plasmid permissiveness (or plasmid uptake ability) among the top 27 abundant OTUs in the transconjugant pools under exposure to artificial sweeteners or not. These OTUs were identified across all transconjugant pools and recipient pools. Results show that all artificial sweeteners enhanced plasmid permissiveness to half of these OTUs (Figure 4). Notably, all Bacteroidota OTUs were increasingly permissive in the presence of artificial sweeteners. For example, the relative permissiveness of *Muribaculaceae* to the pKJK5 plasmid was increased by 3.9- (SAC), 2.1- (SUC), 3.3- (ASP), and 8.2-fold (ACE-K), respectively. Three Actinobacteriota OTUs responded similarly (increased permissiveness) to the exposure of artificial sweeteners. Interestingly, not all Firmicutes OTUs exhibited similar plasmid uptake abilities. Ten of these Gram-positive OTUs such as *Lactobacillus* and *Staphylococcus* became increasingly permissive, while OTUs classified as *Allobaculum* responded with a declined permissiveness in the presence of artificial sweeteners. Among Proteobacteria OTUs that are phylogenetically close to the donor, *Acinetobacter* expectedly responded with an increased permissiveness, but *Escherichia* displayed little permissiveness modulation under exposure to artificial sweeteners. Taken together, artificial sweeteners could increase the plasmid permissiveness to the microbiomes such as Bacteroidota and Actinobacteriota that are commonly distributed in the human gut, and could also increase the plasmid uptake ability of both Gram-negative and Gram-positive OTUs.

## Discussion

The increasing consumption of artificial sweeteners applied in food and beverages has been associated with considerable dysbiosis in gut microbiome composition.^19,33^ This perturbation together with the antimicrobial properties^34^ suggests that these sweeteners could play antibiotic-like roles. Antibiotics have been recognized to promote the specific evolution of antibiotic resistance, trigger the emergence of antibiotic resistance and promote the spread of antibiotic resistance,^35–37^ consequently compromising the efficiencies of drug therapy targeted on pathogenic infections. Our previous study reported that four commonly consumed artificial sweeteners including saccharin (SAC), sucralose (SUC), aspartame (ASP), and acesulfame potassium (ACE-K) can promote horizontal transfer of ARGs across bacterial genera.^26^ However, their effects on the spread of ARGs among gut microbiome that contains diverse bacterial species remain still unknown. In the present study, we established a conjugation model by using mice fecal bacteria as the recipient. We found that these four sweeteners can significantly stimulate the spread of ARGs among both gut microbial communities. Such phenomenon was also confirmed among a nosocomial pathogen *K. pneumoniae*.

Human gut microbiota is more diverse and consists of both Gram-negative and Gram-positive bacteria, and it plays major roles in human health. Horizontal transfer of resistance genes between these microbial flora experience phylogenetic barriers, which reduce gene transfer rate and specifically regulate such genetic exchange among phylogenetically close bacteria.^38^ Our study reported that the tested sweeteners significantly promoted conjugative plasmid transfer from exogenous *E. coli* to both phylogenetically close bacteria (e.g., *Escherichia*) and phylogenetically distant bacteria including *Acinetobacter* and *Staphylococcus*. This indicates that these sweeteners could help donor-recipient couple overcome phylogenetic barriers to exchange ARGs. Specifically, artificial sweeteners could increase the mating pair stabilization (MPS), which is responsible for type IV pilus system required by conjugation process.^39^ This is because we have demonstrated that these sweeteners can up-regulate the expression of MPS-related genes.^26^ The elevated plasmid transfer in gut microbiomes could also indirectly be a consequence of microbiota composition alterations that occur when exposing to artificial sweeteners,^19^ causing different intrinsic responses of bacteria to genetic exchange. Our work found that after 24-h treatment, SAC may alter the gut microbiota composition or diversity, while other sweeteners did not induce such effects. Nevertheless, these results cannot rule out that shifts in gut microbiota composition are necessary for plasmid transfer because of the short-term effects explored in our work. Actually, significant changes in gut microbiomes can be generally observed after several weeks treatment.^17,19,40^ Interestingly, SAC did not but ACE-K induced an inter-individual variation in transconjugant community. Further studies (e.g. long-term exposure) are needed to unravel why the sweeteners could probably shift the gut microbiota composition.

Our results also highlight the gut microbial genera that ARG transfer could preferably be transferred to. We found that gut microbial genera exhibiting a closer phylogenetic distance to the donor bacterium are the most abundant species in transconjugant pools and that artificial sweeteners can promote plasmid transfer within these genera. This is consistent with a previous study that the successful transfer of conjugative plasmid between phylogenetically close bacteria can be easier (in terms of transfer ratio) than phylogenetically distant bacteria.^38^ We also found that artificial sweeteners can promote the plasmid uptake ability of other phylogenetic species including genera *Acinetobacter, Enterococcus* and *Staphylococcus*. These sweeteners could also induce new assortments of species (e.g., *Olsenella* and *Odoribacter*, belonging to Actinobacteriota phylum) that actively receive exogenous plasmid. Moreover, the decline in the abundance of Bacteroidota, a commensal phylum of gut microbiota, in transconjugant pool treated with artificial sweeteners may be accounted for the spread of antibiotic resistance in the gut microbiomes. This is because the decline in Bacteroidota could impair the gut immune response (i.e., indole-3-acetic acid production) and then provide a niche for the development of antibiotic tolerance.^17^ Our previous work has demonstrated that artificial sweeteners can play antibiotic-like roles such as activation of efflux pumps, which can also reduce bacteria susceptibility to antibiotics. This together with the possible enhancement of ARG spread among human gut pathogens would unbalance the gut microbiota composition.

Moreover, our results also provide mechanical insights into the conjugative transfer of plasmid between the donor and the initial fecal community under exposure to artificial sweeteners. The tested sweeteners induced overproduction of ROS, which has been demonstrated to be critical for the conjugation process.^26,41,42^ Specifically, ROS overgeneration increases ROS detoxification and correspondingly stimulates DNA repair system in bacteria, or the SOS response. This response to DNA damage increases the expression of genes for conjugative transfer and hence the conjugation rate. Our previous study has ascribed the enhanced conjugative transfer of ARGs to the ROS overgeneration.^26^ After quenching the generated ROS, the conjugation ratio dramatically declined. It should be noted that the elevated ROS production by the sweeteners could be occurred under hypoxic conditions in our study. This could explain why the sweeteners could also promote plasmid permissiveness among diverse gut microbiomes. Such result is also consistent with the increasing transfer rates of conjugative plasmid TP114 under hypoxic conditions (similar to the intestinal tract).^43^ Although the gut environment is more likely anaerobic, there is the oxygenated zone at the gut mucosa.^44^ Thus, it is still too difficult to rule out whether ROS would be induced by the sweeteners in the gut. In addition, other factors such as cell membrane permeability could also affect horizontal transfer of ARGs^25,26^ and consequently alter the gut microbiota composition. Moreover, artificial sweeteners may induce the production of other free radicals without oxygen involved, probably pose stress on bacteria, and would promote ARG spread among the gut microbiota. Further studies are needed to explore this possibility. Interestingly, we found that some identified anaerobic bacteria such as *Bifidobacterium* and *Faecalibaculum* were also increasingly permissive to the conjugative plasmid (Figure 4). In addition to the spread of antibiotic resistance, overproduction of ROS could also increase antibiotic tolerance in bacteria (because of the collapse of TCA cycle and lower ATP),^45^ which seems consistent with the decline in Bacteroidota transconjugant abundance induced by the sweeteners. Collectively, exposure to artificial sweeteners could reduce the susceptibility of gut microbiome to antibiotic drugs. Note that in this study we cannot rule out that some of the gut bacteria may express high level of *lacI* operon and could probably not express GFP fluorescence even if they successfully receive the plasmid. This will underestimate the transfer of plasmid-borne ARGs in the gut microbiota.

*K. pneumoniae* is a Gram-negative bacterium and is a common pathogen for nosocomial infection.^31^ It has been detected in human lung, blood, and even in gastrointestinal tract,^21,31,46^ where is a huge reservoir of ARGs. Here we found that artificial sweeteners dramatically promoted conjugative transfer of a broad-host-range RP4 plasmid to *K. pneumoniae*, with up to 13.1 folds increased in transfer ratio. This fold increase was obviously larger than the fold changes (2.1 ~ 8.7) induced by the commonly used antibiotics such as ampicillin, cefotaxime and ciprofloxacin.^47^ Thus, exposure to artificial sweeteners could facilitate the evolution of *K. pneumoniae* into multi-drug resistant bacteria. This result also probably demonstrate that artificial sweeteners could promote ARG spread among human gut pathogens.

In conclusion, we show that *in vitro* exposure to artificial sweeteners promotes the dissemination of plasmid-mediated antibiotic resistance in gut microbiota. It should be noted that our experimental conditions are not completely anaerobic and cannot fully represent the real gut environment. Thus, further *in vivo* studies of antibiotic resistance spread in the gut microbiota by adopting more clinically relevant plasmids will be required to validate the antibiotic-like roles of artificial sweeteners. As well, more replicates of samples for sequencing should be prepared to provide the data with a statistical significance and in particular to quantify the fractions of gut microbiota that acquire the plasmid. In addition to antibiotic resistance, the gut microbiomes also carry the innate antimicrobial peptides (responsible for the innate immune defense^48,49^) resistance. It will be also necessary to comprehensively elucidate long-term effects of artificial sweeteners on the horizontal transfer of antimicrobial peptides resistance among the host gut bacteria.

## Methods

### Bacteria strains, plasmids, growth conditions, and non-nutritive sweeteners

Strains and plasmids used in this study are listed in Table S1. Cells were grown at 37°C in Luria broth Miller (LB) broth or LB agar supplemented with the antibiotics when needed at the following working concentrations: kanamycin (Kan) 100 *μ*g/mL and streptomycin (Str) 100 *μ*g/mL.

### In vitro conjugation experiment

In this study, an *in vitro* conjugative transfer model by using mice feces as the recipient was established to comprehensively evaluate the plasmid permissiveness among gut microbial communities and whether artificial sweeteners could promote such permissiveness. Feces were collected from 6- or 8-week-old C57BL/6 mice housed with food and water in the animal facility of the Institute for Molecular Bioscience (Australia). Fecal bacteria were not cultured in the growth medium. The collected feces were directly immersed in PBS (0.1 g/mL) and were homogenized by a vortex (maximal speed, 5 min).^50^ The homogenates were centrifuged at 500 × g for 30 s to remove larger debris.^43^ *E. coli* K-12 MG1655 carrying the IncP-1ε broad host range plasmid pKJK5^51^ was used as donor strain. The donor chromosome (at *att*Tn7 site) was specifically integrated with the Tn7 *lacI^q^-pLpp-mCherry-Km^R^* region, which enables the donor to encode constitutive red fluorescence.^52^ A *gfpmut3b* gene was inserted into the plasmid pKJK5 and was under the control of a promoter operator repressed by *lacI^q^* operon. In this case, the donor strain only expresses red fluorescence (Figure 1a). Once successful transfer of plasmid pKJK5 to a fecal bacterium, *gfp* expression is de-repressed and enables the new host to be green-fluorescent cells. Based on such fluorescent reporters, fecal bacteria that successfully receive plasmid pKJK5 (becoming transconjugants) can be sorted by a fluorescence-activated cell sorting (FACS) and detected by a confocal fluorescence microscopy, respectively. The donor strain was overnight cultured in LB media supplemented with 100 *μ*g/mL of Kan, centrifuged, washed and resuspended in PBS. The cell density was adjusted for mating assays following the previous method.^26,53^ The mixture was then exposed to different concentrations (0, 3, 30, and 300 mg/L) of four commonly used artificial sweeteners (SAC, SUC, ASP, and ACE-K). The highest dose (300 mg/L) is within the FDA recommended acceptable daily intake (ADI) in humans (SAC 15 mg, SUC 5 mg, ASP 50 mg, and ACE-K 15 mg; per kg (bodyweight)). Therefore, the concentrations tested in this study were gut relevant. After mating 24 h at 37°C in the darkness, the samples were analyzed by a flow cytometer to quantify the transfer ratio. We have demonstrated that the tested sweeteners at their all dosages did not impact suppression of *gfp* expression in the donor strain (Figures S11 and S12) and that the *gfp* expression can be stably expressed once the recipient cells acquired the *gfp*-encoded plasmid (Figure S1). Conjugation ratio was calculated with the ratio of transconjugant number and recipient number. Fold changes in conjugative transfer were also calculated by normalization of the transfer ratio to the control group (without sweetener treatment).

To further validate the roles of the tested artificial sweeteners in plasmid permissiveness, we used *E. coli* K-12 LE392 that carries RP4 plasmid (containing ARGs such as *bla_TEM_* and *tetA*) as the donor,^26^ and *K. pneumoniae* ECL8^54^ as the recipient (Figure 1e). The total recipient (includes transconjugants) was enumerated on the plates that contain 100 *μ*g/mL of Str. The number of transconjugants was counted on the plates that contain 100 *μ*g/mL of Kan and 100 *μ*g/mL of Str.

All above samples were prepared in biological triplicate and technical duplicate. A series of analyses such as plasmid extraction, PCR assays and gel electrophoresis, as well as the inhibitory concentrations (ICs) of relevant antibiotics were conducted to further verify the successful transfer of conjugative plasmids from the donor to the recipient. Details are described in Texts S1 and S2.

### Conjugative transfer quantification and imaging analysis

Conjugation events from *in vitro* conjugation were quantified by a CytoFLEX S flow cytometer (Beckman Colter, USA) with excitation at 488 nm and emission at 525 nm (*gfpmut3b*).^26^ Detection results were analyzed by FlowJo 7.6. Transconjugants were sorted by setting up triple gates: the gate of forward scatter-H vs side scatter-H plot was initially set up to focus the particles with bacteria size; the gate of forward scatter-H vs forward scatter-W plot was used to target on singlet; the third gate of 561_TexaRed-A vs 488_SYBR-A plot was used to exclude any auto-fluorescent particles from fecal samples and at the same time sort only out transconjugants (Figure S1). Method of triple-gated sorting was confirmed by both negative (nonfluorescent *E. coli* K-12 MG1655 and the donor strain) and positive (transconjugant *E. coli* K-12 MG1655 with pKJK5 plasmid, generated by mating the nonfluorescent *E. coli* K-12 MG1655 and the donor strains) control samples. The recipient number was also quantified as the event number detected on the down left quadrant. Given that in PBS medium, the bacterial populations do not replicate and the number of donor/ recipient strains did not change after 24 h mating (Figures S5 and S13), the detected number of transconjugant in our study could rule out vertical gene transfer. As well, our previous results have reported that there is no difference of the growth of *E. coli* K-12 MG1655 (donor) under exposure to the sweeteners or not. Therefore, exposure to the sweeteners did not select transconjugants from the mixture.^26^ In the present study, the conjugative transfer ratio was calculated as the transconjugant number divided by the total recipient number. To rule out the effects of the sweeteners on bacterial autofluorescence, we prepared the background controls that consisted of fecal bacteria and the sweeteners, for each conjugation test. Over 300,000 events in total were detected and were analyzed for all the samples.

To confirm the difference in conjugative transfer from the control and treated groups, conjugation events were also visualized by a confocal laser scanning microscopy (ZEISS LSM 710, AxioObserver, Germany).^26,55^ Thresholds of both red and green channels were manually adjusted to exclude any background red or green fluorescence from fecal samples. After that, each mating sample was uploaded for imaging analysis at the fixed thresholds.

### Cellular reactive species detection

Cellular reactive species production in donors and the initial fecal community was measured with reactive oxygen and/or nitrogen species (ROS/RNS) detection assay kit (ab139473), following the manufacturer’s protocol. Typically, bacterial cells were overnight incubated in fresh LB media containing corresponding antibiotics or not. After that, cell pellets were collected by centrifugation, washed and were re-suspended in PBS. The cell pellet was resuspended in 100 *μ*L of ROS/RNS 3-Plex detection mix (NO, superoxide and total ROS detection reagent). After 2 h periodic shaking incubation at 37°C, the cells were centrifuged at 400 × g for 5 min to remove mix reagent residues. This is followed by treatments (in PBS) with various concentrations of artificial sweeteners. After 30 min induction, the production of each reactive species was detected by a CytoFLEX S flow cytometer (Beckman Colter, USA). Both positive (inducers for reactive species production), negative (inhibitors for reactive species production) and non-stained bacteria samples were also parallelly prepared. Fecal bacteria were tested with the initial concentration of 0.001 g/mL. The initial cell density of other pure cultures was around 10^6^ CFU/mL. The effect of artificial sweeteners on bacterial autofluorescence was ruled out by establishing the background control (without staining) for each ROS measurement.

### Cell sorting and 16S rRNA gene amplicon sequencing

After 24 h mating, samples treated with or without 300 mg/L of each sweetener were sorted by FACS (BD FACSAria III, USA). All samples were diluted at least 10 folds with PBS before sorting. Transconjugants were gated and sorted based on bacterial size, green fluorescence and exclusion of red fluorescent donor cells (Figure S14).^52^ At least 50,000 transconjugant events were sorted from each sample. Sorted transconjugant cells were stored at −80°C until lysis and 16S rRNA gene amplicon sequencing analysis. In addition, the recipient (fecal bacteria) samples were also prepared with or without 300 mg/L of each sweetener treatment. Bacterial DNA from transconjugants and recipients were extracted by the Qiagen DNeasy Powersoil Pro Kit (Qiagen #47016), following the manufacturer’s protocols (Text S3). High-throughput sequencing of the V3 and V4 regions of the bacterial 16S rRNA gene was performed by the Australian Center for Ecogenomics (ACE), where the V3-V4 regions were targeted and amplificated using the 341 F forward (5’-CCTACGGGNGGCWGCAG-3’) and 806 R reverse (5’- GACTACHVGGGTWTCTAATCC −3’) primers. The PCR amplicons were purified using Agencourt AMPure XP beads (Beckman Coulter). The 16S rRNA library was constructed by following Illumina workflow (# 15044223 Rev. B). All libraries passed the quality control and were sequenced on MiSeq Sequencing System (Illumina, V3 300bp paired-end reads). The resulting sequencing reads were provided as demultiplexed fastq files for the following analysis.

### Sequencing data analysis

The 16S rRNA amplicon sequencing analysis was conducted using QIIME2 (https://qiime2.org).^56^ Raw amplicon sequencing reads were processed (filtered, trimmed, dereplicated and merged) with DADA2 to generate amplicon sequence variants (ASVs), in order to denoise operational taxonomic unit (OTU) reads. The inferred ASVs were then blasted from SILVA database (https://www.arb-silva.de).^57^ The sequencing data for each sample were normalized by the samples with the smallest amount of data. High-quality sequences of 97% similarity were clustered into OTUs. Statistical analysis of microbial community diversity was performed by the Calypso software V8.84 (http://cgenome.net/calypso).^58^ Bray-Curtis PCoA was conducted at OTU level. Phylogenetic trees of transconjugal pool were plotted using iToL (https://itol.embl.de)^59^ and tree clustering of OTUs was performed using MEGA version X.^60^

### Plasmid permissiveness analysis at OTU level

To estimate the potentially horizontal transfer of conjugative plasmid pKJK5 among fecal bacteria community, we analyzed plasmid permissiveness in the recipient communities at OTU level.^61^ Typically, the apparent OTU-level permissiveness was initially calculated as the ratio of relative transconjugant and recipient abundances after the mating incubation. The relative change in plasmid permissiveness of each OTU was then calculated by the normalization of permissiveness from sweetener-treated groups to permissiveness from the control group. The value of relative change in plasmid permissiveness larger or lower than 1 would suggest an increase or decrease in plasmid permissiveness of fecal bacteria species in the presence of artificial sweeteners, respectively.

### Statistics

Experiments of conjugation (plating, flow cytometry, and ICs measurement) and ROS measurement were conducted independently at least in biological triplicate. The data were expressed as mean ± standard deviation and were analyzed with SPSS 27.0 (SPSS, Chicago, USA). The phenotypic results were analyzed by Independent-sample *t* test method, with the Bonferroni adjustment.^62^ *P* values less than 0.05 are considered to be statistically significant. No statistical analysis was conducted for the 16S rRNA sequencing data (*n* = 1).

## Supplementary Material

Supplemental Material

## Data Availability

All 16S rRNA gene amplicon sequencing raw data were deposited in the National Centre for Biotechnology Information (NCBI) Sequence Read Archive (SRA; https://dataview.ncbi.nlm.nih.gov/object/PRJNA759753) under BioProject number PRJNA759753.
