# Supplementary material for: Non-caloric artificial sweeteners modulate conjugative transfer of multi-drug resistance plasmid in the gut microbiota
Source: Gut Microbes. 2022 Dec 16;15(1):2157698. doi: 10.1080/19490976.2022.2157698 (PMC9762752; doi:10.1080/19490976.2022.2157698)
Supplement: Supplemental Material [file KGMI_A_2157698_SM8621.docx]

**Supplementary Information**

Non-caloric artificial sweeteners modulate conjugative transfer of multi-drug resistance plasmid in the gut microbiota

Zhigang Yu^a^, Ian R. Henderson^b^, Jianhua Guo^a^*

*^a^Australian Centre for Water and Environmental Biotechnology (formerly AWMC), The University of Queensland, St. Lucia, Brisbane, QLD 4072, Australia*

*^b^Institute for Molecular Bioscience, The University of Queensland, St. Lucia, Brisbane, QLD 4072, Australia*

* Corresponding author, Email: [jianhua.guo@uq.edu.au](mailto:jianhua.guo@uq.edu.au)

This file contains

Texts S1 to S3

Tables S1 to S2

Figures S1 to S14

References

**Text S1. Plasmid extraction and PCR assays of antibiotic resistance genes (ARGs)**

In Model I, five transconjugant colonies were randomly selected from the selective plates and were overnight incubated in LB media that contained corresponding antibiotics. Plasmids from the collected strains were extracted by GeneJET Plasmid Miniprep Kit (Life Technologies, Australia), according to the manufacturer’s instructions. After that, the presence of plasmid in transconjugant was initially confirmed by the agarose gel electrophoresis. As well, both donor and recipient were also used as positive and negative controls. Meanwhile, the extracted plasmid was also used for the detection of antibiotic resistance genes such as *bla_TEM_* and *tetA* by a qualitative PCR technique [1]. The 20 *μ*L volume reaction assays, which consists of 1 *μ*L of DNA sample, 10 *μ*L of Platinum Green Hot Start PCR 2 × Master Mix (Invitrogen by Thermo Fisher Scientific), 2 *μ*L of Platinum GC Enhancer, 6.6 *μ*L of Milli-Q water, 0.2 *μ*L of forward and reverse primers for *bla_TEM_* or *tetA*, were carried out. The procedure was programmed as: 94 ℃ for 10 min, followed by 30 cycles at 94 ℃ for 1 min, at 60 ℃ for 1 min, at 72 ℃ for 1 min, and a final extension at 72 ℃ for 7 min. The amplification process was run by Applied Biosystems.

In Model II, plasmid extraction from sorted transconjugants was also performed following the similar methods. Considering that mice fecal bacteria community could contain some unknown plasmids, gel electrophoresis of extracted plasmid failed to distinguish the successful transfer of pKJK5 plasmid. Alternatively, PCR assays of both *mCherry* and *gfpmut3b* genes enabled to confirm such transfer process. The same procedures of PCR conditions (described above) were adopted for *mCherry*. PCR amplification of *gfpmut3b* was also conducted using the similar procedures but at different annealing temperature (51 ℃).

After plasmid extraction and PCR assays of ARGs, visualization of all bands was performed by SYBR safe DNA gel staining and the GeneRuler 1 kb DNA ladder.

**Text S2. Measurement of inhibitory concentrations of antibiotics**

Colonies from transconjugant selective plates were used for the measurement. After overnight incubation in LB media that contained corresponding antibiotics, cell pellets were collected by centrifugation, washed and resuspended in PBS solution. After that, cell suspensions were loaded onto 96-well plates. Each well was 150 *μ*L in total and consisted of different concentrations of antibiotics. The final cell density was approximately 10^5^ CFU/mL. The plates were overnight incubated at 37 ℃ and the optical density at 600 nm wavelength was then measured by a CLARIOstar Multimode plate reader (BMG LABTECH). All samples were prepared in biological triplicate. IC90, determined as the antibiotic concentration, at which 90% of cell growth is inhibited, was used in this study.

**Text S3. DNA extraction**

DNA was extracted from 50 - 200 mg of raw sample with a preliminary step of bead beating using 0.1 (in diameter) glass beads (BioSpec Products #11079101) on the Powerlyser 24 homogenizer (Mo-Bio #13155). Sample was added to a bead tube filled with 850 *μ*l of CD1 (Qiagen cat #47016) and vortexed to mix. The tubes were heated at 65 ℃ for 10 min. The sample was then bead beat for five minutes at 2000 RPM then centrifuged for one minute at 15,000g. The resulting lysate was transferred to a new collection tube. Extraction was as per Qiagen DNeasy Powersoil Pro Kit (cat #47016). Final elution volume is 50 *μ*l.

Table S1. Strains used in this study

| Strains | Genotype/description | Source |
| --- | --- | --- |
| *E. coli* K-12 MG1655 | No plasmids; contains chloramphenicol resistance on chromosome | [1] |
| *E. coli* K-12 MG1655/*gfp*-pKJK5 | The host chromosome is tagged with *mCherry* gene; plasmid is labelled with *gfp* gene and contains resistance genes (kanamycin^R^ and trimethoprim^R^) | [2, 3] |
| *E. coli* K-12 LE392 | Contains RP4 plasmid that carries resistance genes (ampicillin^R^, kanamycin^R^, and tetracycline^R^) | [1] |
| *Klebsiella pneumoniae* ECL8 | Carries multiple resistance including ampicillin^R^, chloramphenicol^R^, tetracycline^R^, and streptomycin^R^, but is susceptible to kanamycin | [4] |
| *E. coli* K-12 MG1655 -pMS6198A | Plasmid carries multiple resistance including ampicillin^R^ and kanamycin^R^ but not streptomycin^R^ | [5] |

Table S2. PCR primers used in this study

| Primer | Amplicon size | Sequence (5′-3′) |
| --- | --- | --- |
| *bla_TEM_* Forward | 861 bp | TTACCAATGCTTAATCAGTGAGGC |
| *bla_TEM_* Reverse |  | ATGAGTATTCAACATTTCCGTGTCG |
| *tetA* Forward | 1200 bp | CGTGTATGAAATCTAACAATGCGCT |
| *tetA* Reverse |  | CCATTCAGGTCGAGGTGGC |
| *mCherry* Forward |  | GCCATCATCAAGGAGTTCAT |
| *mCherry* Reverse |  | ATGGTGTAGTCCTCGTTGTG |
| *gfpmut3b* Forward |  | ATATAGCATGCGTAAAGGAGAAGAACTTTTCA |
| *gfpmut3b* Reverse |  | CTCTCAAGCTTATTTGTATAGTTCATCCATGC |

**
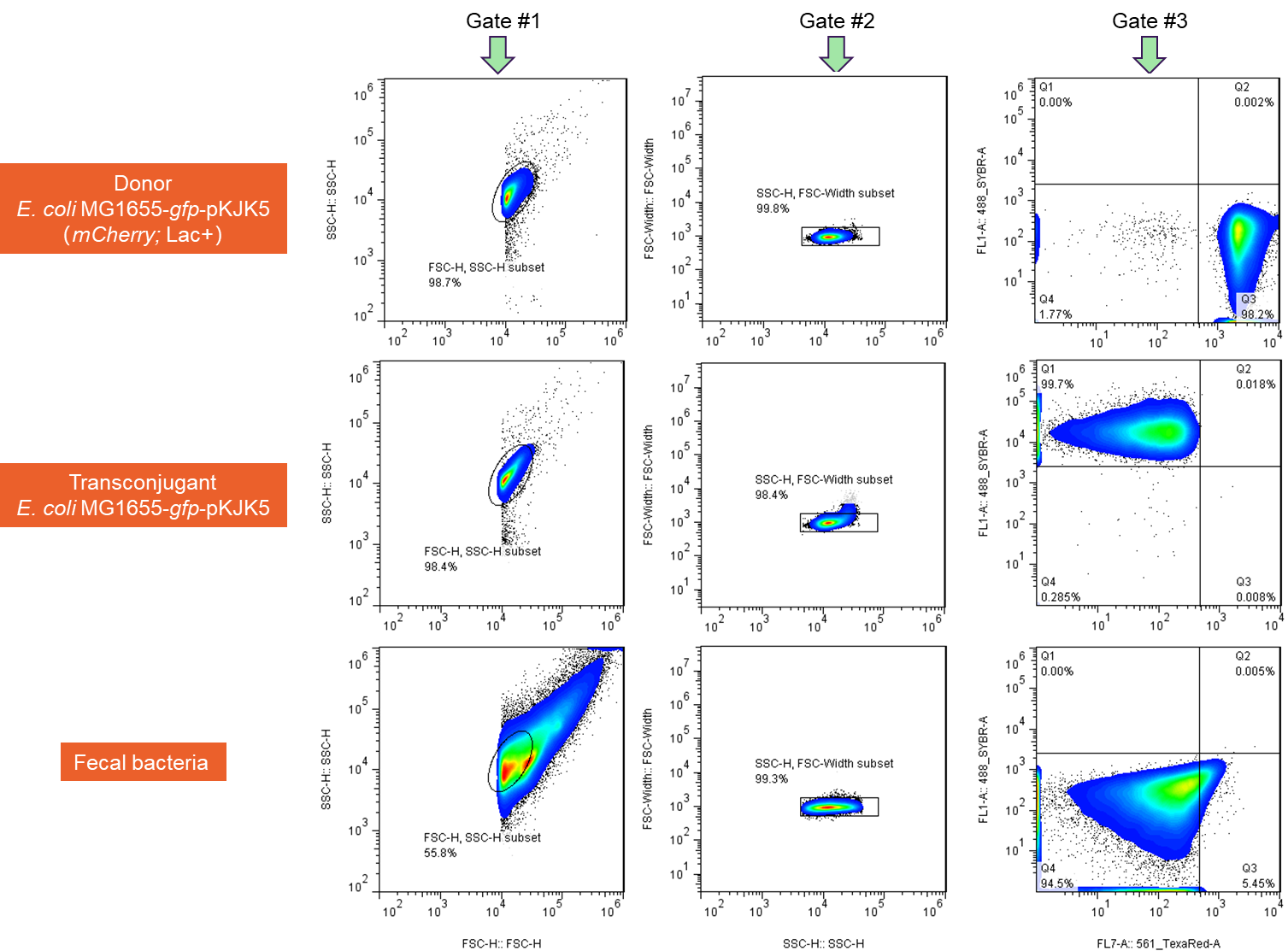
**

**Figure S1.** FACS sorting of red-, green-, and non-fluorescent cells. Gate I sorts for bacterial size based on forward scatter-H and side scatter-H plot; Gate II sorts for singlet based on forward scatter-H and forward scatter-W plot; and Gate III selects only those green cells (left top quadrant). Top panel shows the results from the donor *E. coli* MG1655 carrying *gfp*-pKJK5 plasmid and chromosomally-encoded *mCherry* gene; The middle panel shows the results from positive control (transconjugants); The bottom panel shows the results from fecal bacteria.

**Figure S2.** Fold changes in conjugative transfer between E. coli MG1655 and gut bacteria communities, under exposure to glucose, sucrose, stevia, and NaCl. The used concentration of glucose, sucrose, and stevia was 300 mg/L. Two dosages of NaCl were tested with the same osmoles (1.5 Osmol/L, or 88 mg/L of NaCl) of solute and the same concentration (300 mg/L) to artificial sweeteners, respectively. Significant differences between individual sweetener-treated groups and the control (0 mg/L of sweeteners) were tested with Independent-sample *t* test and the Bonferroni correction; ns, means no significant difference.


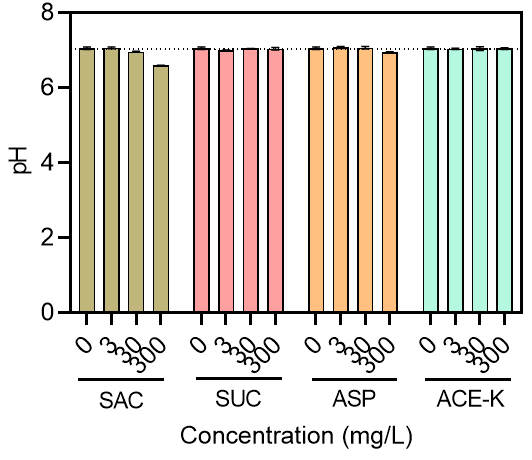


Figure S3. Measurement of culture pH in the presence of artificial sweeteners

**
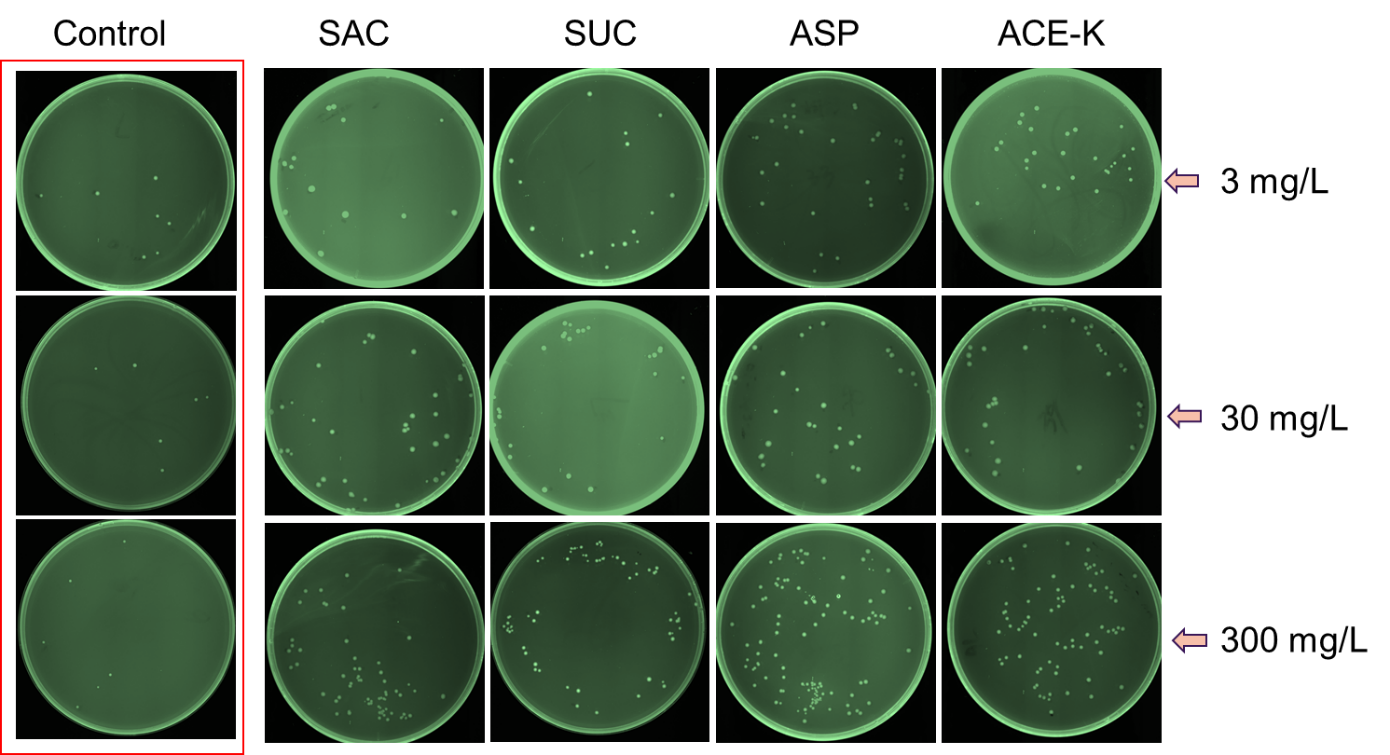
**

**Figure S4.** Images of transconjugant plates showing the number of transconjugants induced by artificial sweeteners. Images were taken by Uvitec UVIDOC HD6 Gel Doc System.

**Figure S5.** The number of recipient *K. pneumonia* ECL8 treated with various concentrations of artificial sweeteners (*N* = 6). Significant differences between individual artificial sweetener-treated groups (3, 30 and 300 mg/L) and the control (0 mg/L of sweeteners) were tested with Independent-sample *t* test and the Bonferroni correction. There is no significant difference (*p* > 0.05) between the control and treated groups.

**Figure S6.** ICs measurement of the donor *E. coli* LE392, the recipient *K. pneumonia* ECL8 and transconjugants (from the control group and 300 mg/L of sweetener-treated groups) against kanamycin and streptomycin

**
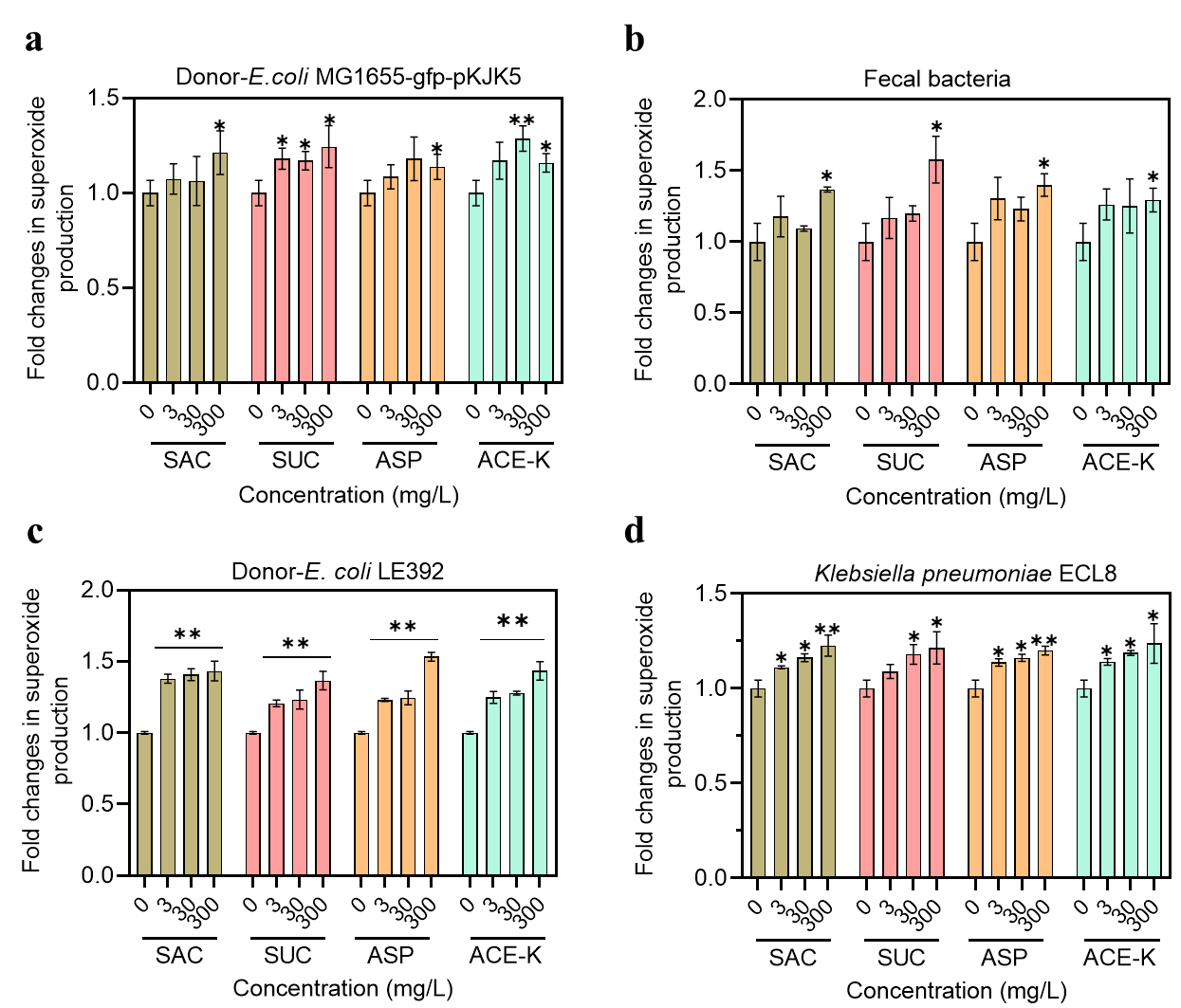
**

**Figure S7.** Generation of superoxide induced by the treatments with artificial sweeteners at different concentrations. **a**, Fold changes in superoxide production in the donor strain *E. coli* MG1655 (*gfp*-pKJK5). **b**, Fold changes in superoxide production in mice fecal bacteria as the recipient. **c**, Fold changes in superoxide production in the donor strain *E. coli* LE392 (RP4); **d**, Fold changes in superoxide production in the recipient strain *K. Pneumonia* ECL8. Significant differences between individual sweetener-treated groups and the control (0 mg/L of sweeteners) were tested with Independent-sample *t* test and the Bonferroni correction, * *p* < 0.05 and ** *p* < 0.01.

**Figure S8.** Fold changes in NO radical production in the donor *E. coli* LE392. Significant differences between individual sweetener-treated groups and the control (0 mg/L of sweeteners) were tested with Independent-sample *t* test and the Bonferroni correction, ns means *p* > 0.05.

**
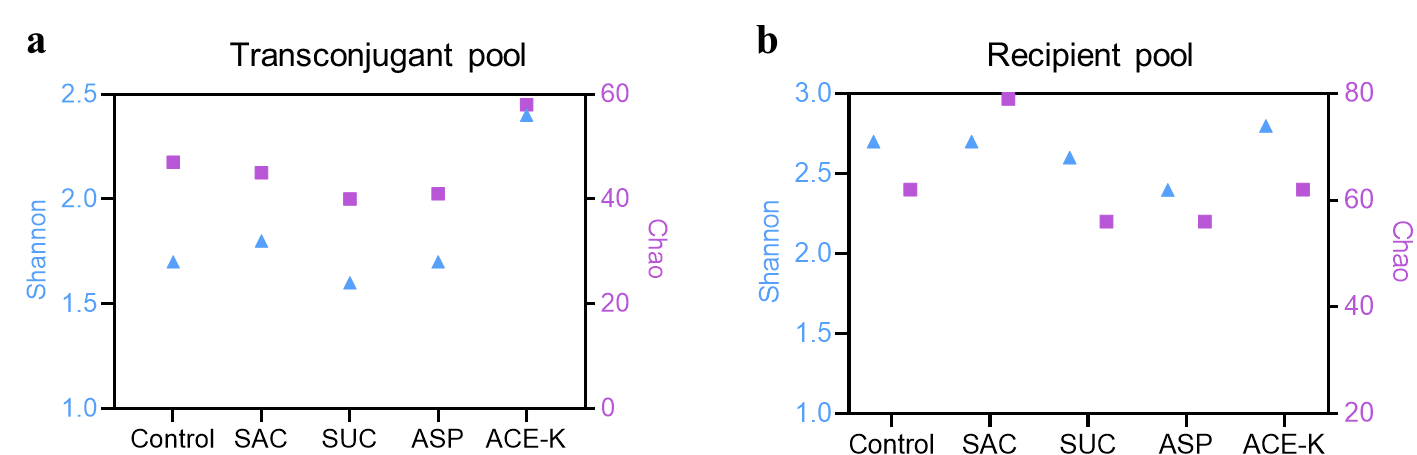
**

**Figure S9.** Shannon index (left Y axis) and Chao index (right Y axis) of transconjugant (**a**) and recipient (**b**) pools

**Figure S10.** Relative abundance of four top abundant phyla in transconjugant pools for different conditions


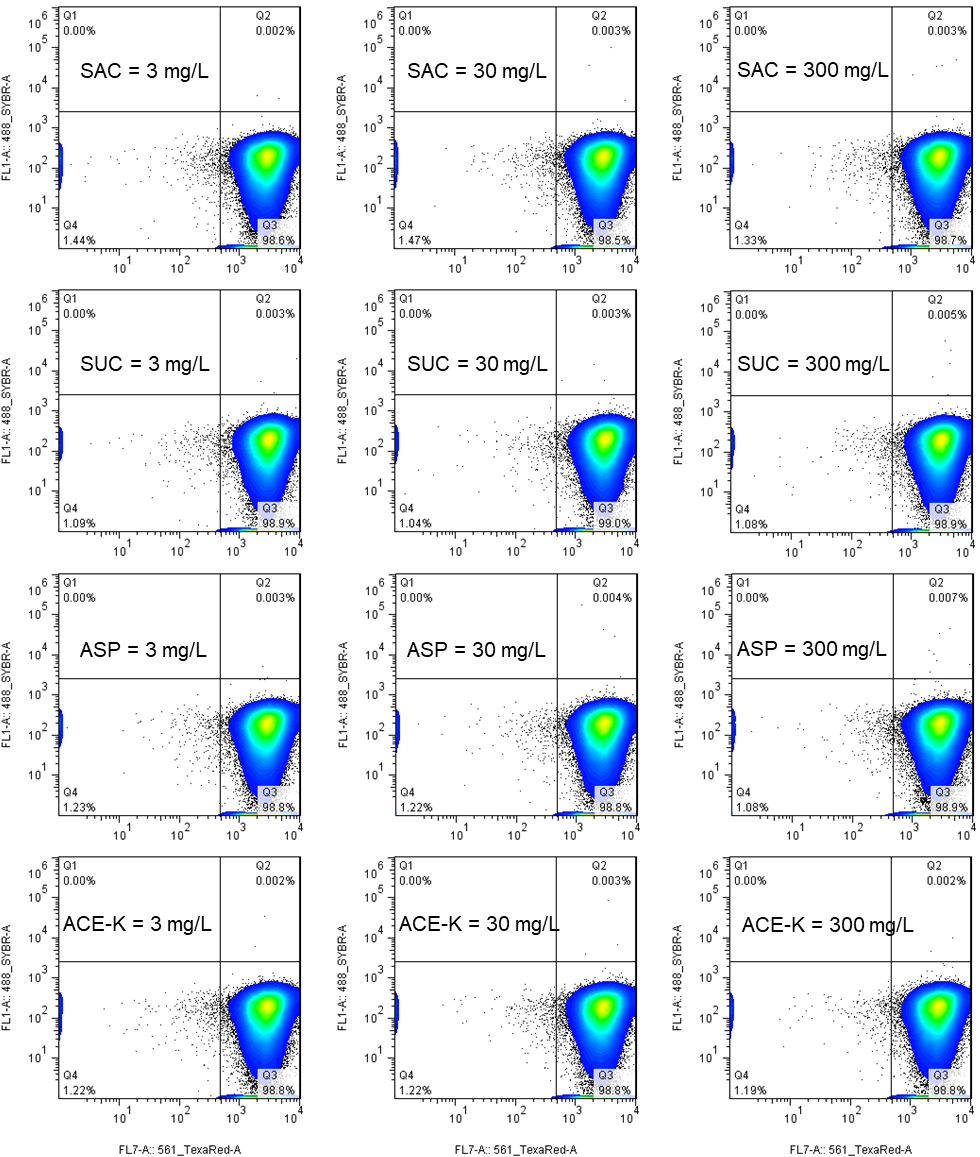


**Figure S11.** Flow cytometry analysis of green fluorescence in the donor under exposure to artificial sweeteners

**Figure S12.** Quantitative PCR analysis of *gfp* gene expression in the donor under exposure to different concentrations of artificial sweeteners. Each sample was prepared in biological triplicate. Significant differences between individual sweetener-treated groups and the control (0 mg/L of sweeteners) were tested with Independent-sample *t* test and the Bonferroni correction; ns, means no significant difference.

**
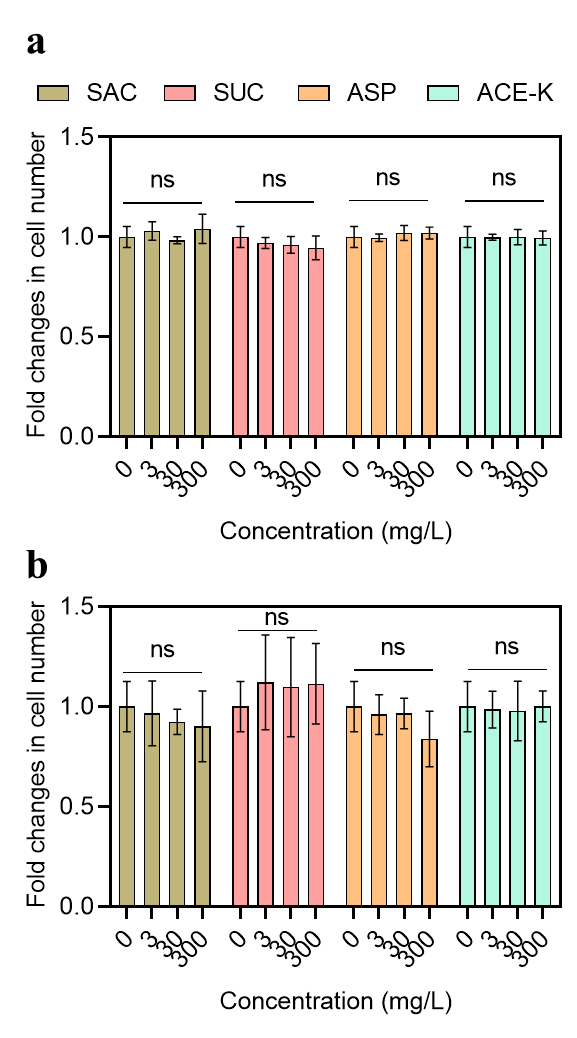
**

**Figure S13.** Changes in the number of both donor *E. coli* MG1655 (*gfp*-pKJK5) (**a**) and fecal bacteria (**b**) after 24-h conjugation assay. Significant differences between individual sweetener-treated groups and the control (0 mg/L of sweeteners) were tested with Independent-sample *t* test and the Bonferroni correction; ns means no significant difference.

**
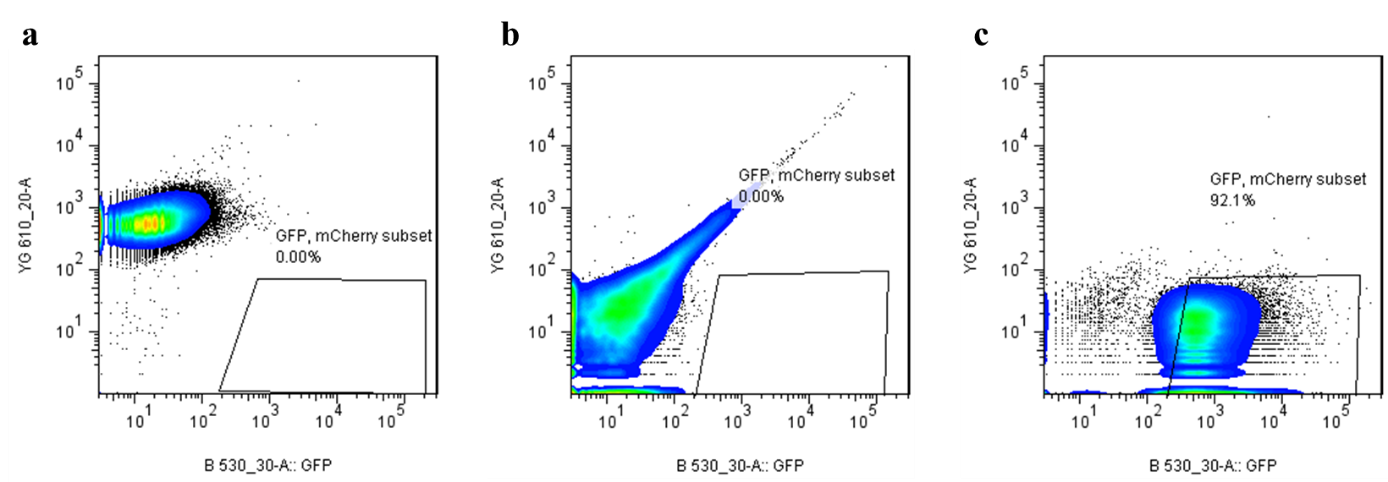
**

**Figure S14.** FACS sorting of green-fluorescent cells from (**a**) the donor *E. coli* MG1655 carrying *gfp*-pKJK5 plasmid and chromosomally-encoded *mCherry* gene, (**b**) fecal bacteria, and (**c**) transconjugant *E. coli* MG1655 carrying *gfp*-pKJK5 plasmid, respectively. The area selected in each panel represents for green-fluorescent cells and was used for cell sorting of transconjugants from the mating system.

**References**

1. Yu Z, Wang Y, Lu J, Bond PL, Guo J. Nonnutritive sweeteners can promote the dissemination of antibiotic resistance through conjugative gene transfer*.* ISME J. 2021; 15: 2117-2130.

2. Bahl MI, Hansen LH, Goesmann A, Sørensen SJ. The multiple antibiotic resistance IncP-1 plasmid pKJK5 isolated from a soil environment is phylogenetically divergent from members of the previously established α, β and δ sub-groups*.* Plasmid 2007;58:31-43.

3. Klümper U, Riber L, Dechesne A, Sannazzarro A, Hansen LH, Sørensen SJ, et al. Broad host range plasmids can invade an unexpectedly diverse fraction of a soil bacterial community*.* ISME J. 2015;9:934-945.

4. Forage R, Lin E. DHA system mediating aerobic and anaerobic dissimilation of glycerol in *Klebsiella pneumoniae* NCIB 418*.* J Bacteriol. 1982;151:591-599.

5. Hancock SJ, Phan MD, Peters KM, Forde BM, Chong TM, Yin WF, et al. Identification of IncA/C plasmid replication and maintenance genes and development of a plasmid multilocus sequence typing scheme*.* Antimicrob Agents Chemother. 2017;61.
